# Supplementary material for: Spatial distribution of clinical computer systems in primary care in England in 2016 and implications for primary care electronic medical record databases: a cross-sectional population study
Source: BMJ Open. 2018 Feb 28;8(2):e020738. doi: 10.1136/bmjopen-2017-020738 (PMC5855245; doi:10.1136/bmjopen-2017-020738)
Supplement: Author's manuscript [file bmjopen-2017-020738.draft_revisions.pdf]

BMJ Open is committed to open peer review. As part of this commitment we make the peer review history of every article we publish publicly available.

When an article is published we post the peer reviewers' comments and the authors' responses online. We also post the versions of the paper that were used during peer review. These are the versions that the peer review comments apply to.

The versions of the paper that follow are the versions that were submitted during the peer review process. They are not the versions of record or the final published versions. They should not be cited or distributed as the published version of this manuscript.

BMJ Open is an open access journal and the full, final, typeset and author-corrected version of record of the manuscript is available on our site with no access controls, subscription charges or pay-per-view fees (http://bmjopen.bmj.com).

If you have any questions on BMJ Open's open peer review process please email editorial.bmjopen@bmj.com

BMJ Open

Spatial distribution of clinical computer systems in primary care in England: implications for primary care electronic medical record databases

Journal:	*BMJ Open*
Manuscript ID	bmjopen-2017-020738
Article Type:	Research
Date Submitted by the Author:	20-Nov-2017

Complete List of Authors:	Kontopantelis, Evangelos; University of Manchester, Division of Informatics, Imaging and Data Sciences; Faculty of Biology, Medicine and Health; NIHR School for Primary Care Research, Centre for Primary Care<br>Stevens, Richard; University of Oxford, Nuffield Dept Primary Care Health Sciences<br>Helms, Peter; University of Aberdeen, The Institute of Applied Health Sciences<br>Edwards, Duncan; University of Cambridge, Department of Public Health and Primary Care<br>Doran, Tim; University of York, Department of Health Sciences<br>Ashcroft, Darren; University of Manchester, Division of Pharmacy & Optometry; Faculty of Biology, Medicine and Health

**Primary Subject Heading**:	General practice / Family practice
Secondary Subject Heading:	Epidemiology, Health informatics, Health services research, Research methods
Keywords:	Electronic Health Records, Primary Care Databases, clinical computer systems, EMIS, Vision, CPRD

SCHOLARONE™ Manuscripts

Spatial distribution of clinical computer systems in primary care in England: implications for primary care electronic medical record databases

Evangelos Kontopantelis[1 2], Richard Stevens[3], Peter Helms[4], Duncan Edwards[5], Tim Doran[6], Darren M Ashcroft[1 7].

[1] NIHR School for Primary Care Research; University of Manchester; Manchester, UK.
[2] Division of Informatics, Imaging and Data Sciences; Faculty of Biology, Medicine and Health; University of Manchester; Manchester, UK.
[3] NIHR School for Primary Care Research; Nuffield Department of Health Care Sciences; University of Oxford, Oxford, UK.
[4] The Institute of Applied Health Sciences; University of Aberdeen.
[5] Department of Public Health and Primary Care; School of Clinical Medicine; University of Cambridge; Cambridge, UK.
[6] Department of Health Sciences; University of York; York, UK.
[7] Division of Pharmacy & Optometry; Faculty of Biology, Medicine and Health; University of Manchester; Manchester, UK.

Correspondence to:
Evangelos Kontopantelis
Division of Informatics, Imaging and Data Sciences, University of Manchester
5th floor Williamson Building
University of Manchester
e-mail: e.kontopantelis@manchester.ac.uk

Keywords

For peer review only

Abstract

Objectives

UK Primary Care Databases (PCDs) are used by researchers worldwide to inform clinical practice. These databases have been primarily tied to single clinical computer systems, but little is known about the adoption of these systems by primary care practices or their geographical representativeness. We explore the spatial distribution of clinical computing systems and discuss the implications for the longevity and regional representativeness of these resources.

Design

Cross-sectional study.

Setting

English primary care clinical computer systems.

Participants

7,526 general practices in August 2016.

Methods

Spatial mapping of family practices in England in 2016 by clinical computer system at two geographical levels: the lower Clinical Commissioning Group (CCG, 209 units) and the higher NHS regions (14 units). Data for practices included: numbers of doctors, nurses, and patients; and area deprivation.

Results

Of 7,526 practices, EMIS was used in 4,199 (56%), SystmOne in 2,552 (34%) and Vision in 636 (9%). Great regional variability was observed for all systems, with EMIS having a stronger presence in the West of England, London and the South; SystmOne in the East and some regions in the South; and Vision in London, the South, Greater Manchester and Birmingham.

Conclusions

PCDs based on single clinical computer systems are geographically clustered in England. For example, CPRD and THIN, the most popular primary care databases in terms of research outputs, are based on the Vision clinical computer system, used by less than 10% of practices and heavily concentrated in three major conurbations and the South. Researchers need to be aware of the analytical challenges posed by clustering, and barriers to accessing alternative PCDs need to be removed.

Keywords

Electronic Health Records; Primary Care Databases; clinical computer systems; representativeness; EMIS; Vision; SystmOne; CPRD; THIN; QRESEARCH; ResearchOne.

For peer review only - http://bmjopen.bmj.com/site/about/guidelines.xhtml

Strengths and limitations of the study

- Cross-sectional analysis of all clinical computer systems used in English primary care, in August 2016.
- Data allowed a detailed description of regional use of each clinical computer system at the Clinical Commissioning Group (CCG) level, and the discussion of implications for UK Primary Care Databases (PCDs).
- Although regional presence of a clinical computer system cannot be equated to contribution to a PCD, since contributing practices are anonymised, inferences on the regional representativeness of UK PCDs are still possible.

For peer review only

For peer review only - http://bmjopen.bmj.com/site/about/guidelines.xhtml

Introduction

Primary care in the UK has been almost fully computerised since the early 1990s. [1] Following the introduction of partial subsidies for the purchase of clinical computer systems in 1998 and full subsidies in 2003 (in anticipation of the implementation of a national pay-for-performance programme) UK primary care became fully computerised.[2 3] Interoperability requirements of the National Health Service led to the universal adoption of a loosely hierarchical clinical coding system, known as Read codes,[4] which is due to be replaced in April 2018 by a multi-hierarchical coding system (SNOMED CT).[5] Various commercial providers were permitted to enter the market for clinical computer systems, resulting in numerous different systems with varying interfaces, mechanisms and implementations of Read code usage.[6] By 2010-11, seven clinical computer systems were consistently active in England, holding 99% of the market share: EMIS systems (LV and PC) were active in 54.7% of practices, followed by Vision v3 (18.1%) and SystmOne (17.8%), with the remaining 9.4% held by other systems (Synergy, Practice Manager, Premiere and the then newly launched EMIS Web).[2]

The uniformity and interoperability standards have facilitated the creation of large repositories of primary care electronic health records (EHRs), which contain the complete primary care records of patients attending general practices in the UK. The secondary use of these EHRs by researchers – both within and outside the UK – has been increasing exponentially, [7] and they have provided insights in numerous research areas, including: real-world effectiveness, adverse events, resource utilisation, condition prevalence and incidence, quality of care, and policy interventions.[8] Several EHR databases exist, maintained by the different clinical computer system providers, drawing data from practices using their systems that have agreed to make patient data available for secondary use. The four largest EHR databases (hereafter primary care databases, or PCDs) in terms of numbers of patient records are the Clinical Practice Research Datalink (CPRD), The Health Improvement Network (THIN), QResearch and ResearchOne.

The CPRD (formally General Practice Research Database, GPRD) was established in 1987 and has been owned by the Secretary of State for Health since 1994. In May 2017, the CPRD covered approximately 8% of the UK population, with 718 contributing general practices and over 17 million total patients (historical and current). The CPRD primarily collects data from Vision practices, although it is currently undergoing an expansion to include EMIS practices, and a future expansion to cover SystmOne practices is planned. THIN was established in 2003 as a collaboration between the company owning Vision (In Practice Systems Ltd) and the CSD Medical Research Group (now Quintiles IMS). In April 2015, THIN reported covering 6% of the UK population, with 562 practices and 11 million total patients. There is a considerable overlap (around 60%) between CPRD and THIN practices, which has implications for studies wishing to

For peer review only - http://bmjopen.bmj.com/site/about/guidelines.xhtml

replicate findings between different databases.[9] QResearch collects data from practices using EMIS systems and is the biggest PCD, with approximately 1500 practices in 2017, covering a population of more than 22 million patients.[10] ResearchOne is a collaboration between the provider of SystmOne (TPP) and the University of Leeds, reporting 28 million (primary and secondary care) records and 423 practices in 2017.

The geographical coverage of PCDs is dependent on the location of practices using the parent clinical computer system, which is in turn dependent on historical patterns of market penetration by the software suppliers and system uptake by general practices. Geographical representativeness is an important prerequisite if analysts are to generalise PCD findings to the whole of England and the UK, which is what routinely happens in practice. This is due to great regional variability across England in terms of population characteristics (primarily, age, ethnicity and deprivation),[11] or even regional variation in hard outcomes. For example, a persistent mortality divide between North and the South of England has existed since the middle of the previous century,[12] while, more recently, much higher mortality rates were observed for young adults in the North of England.[13] There is also regional variation in the organisation and productivity of health services in England,[14 15] which could have important implications for the generalisability of health services research with the use of regionally unrepresentative PCDs. Therefore, the aim of this paper is to describe the regional distribution of clinical computer systems in English primary care, evaluate the implications of the current picture of representativeness and provide some insight into the sustainability of existing PCDs.

Methods

Data

Clinical computer system information was obtained from NHS Digital after direct communication, for August 2016. Primary care workforce and patient information as of 30 September 2016 was downloaded from the NHS Digital website.[16] At the practice level, information was available on geography (Clinical Commissioning Group and NHS region), patient list size by age groups, and numbers and full-time equivalent (FTE) for GPs and nurses. Deprivation was quantified using the 2015 release of the Index of Multiple Deprivation (IMD), a complete aggregate measure widely used to quantify area deprivation, attributed to the practice location.[17] Spatial coordinates for NHS organisational units in 2016 were obtained from the ONS open geography portal.[18] We focused on two organisational levels, the lower Clinical Commissioning Groups (CCGs) with 209 units, and the higher NHS regions with 14 units.

For peer review only - http://bmjopen.bmj.com/site/about/guidelines.xhtml

Analyses

For all aspects of data manipulation and analysis we used Stata v14.1. Whenever medians are reported, we also report the 25th and 75th centiles. Spatial maps were plotted using the *spmap* command.[19] Practice-level data were aggregated by clinical computer system, to provide information on all patients, patients aged 75 or over, GPs and nurses, practice location deprivation and list size. Counts for each clinical computer system, by NHS region, were also calculated. Spatial graphs at the CCG level, with additional information on NHS regions, were plotted for the three most popular clinical computer systems, to provide a visual guide in regional distribution and representativeness.

Results

System information was missing for 49 (0.7%) of 7,526 general practices. EMIS systems were used in 4,199 practices (56%), with all but 23 of these using EMIS Web. SystmOne was used in 2,552 (34%), Vision in 636 (9%) and Evolution in 90 (1%) practices. Patterns of area deprivation, based on the locations of general practices, were similar across all systems. SystmOne practices tended to be larger (median of 7,080 patients), followed by EMIS (6,833), Vision (6,279) and Evolution (6,222).

Great regional variability in system usage was observed both at the NHS Region level (Table 1) and CCG level (Figures 1-3). EMIS is present in all but 18 of the 209 CCGs (91.4%), with a much stronger presence in the West of England, London and the South. SystmOne is present in 120 CCGs (57.4%), and is mainly active in the East and some regions in the South. Vision, although with a much lower market share than SystmOne, is still used in 96 CCGs (45.9%), mainly in London, the South, Greater Manchester and Birmingham. Evolution is only present in 18 CCGs (8.6%) and is primarily used in the South West.

<Please place Table 1 here>

<Please place Figure 1 here>

<Please place Figure 2 here>

<Please place Figure 3 here>

Discussion

High regional variability exists in the use of different clinical computer systems in English primary care, which should be a consideration when utilising primary care electronic health databases based on this population in the future, especially if effect heterogeneity (or other forms of heterogeneity) is context relevant. For example, drawing nationwide conclusions in health services organisation would be more problematic than identifying medication side-

For peer review only - http://bmjopen.bmj.com/site/about/guidelines.xhtml

effects. EMIS Web is by far the most widely used clinical computer system and therefore QResearch is the most nationally representative single database – potentially able to collect data from almost all English CCGs. SystmOne has a very strong presence is many parts of England, but no presence in many CCGs in the North West, West Midlands, London and South East. The ResearchOne database is therefore unable to capture data from many regions. Finally, Vision is the most geographically restricted of the three major clinical computer systems, with relatively few practices heavily concentrated in three conurbations and the South. The CPRD and THIN databases are therefore currently unable to provide comprehensive coverage of large parts of the country, particularly in the North and East of England.

Strengths and limitations of the study

The main strength of this study is the use of numerous national administrative datasets of high data quality, allowing us to obtain a complete picture for the whole of England. The main weakness of the work is the fact we cannot equate the regional presence of a clinical system to active contribution to a primary care database – not all practices contribute data and contributing practices are anonymised – and we have therefore discussed potential contribution instead.

Findings and implications

The current picture of clinical system usage in English primary care is very different to what was reported for 2011.[2] Although EMIS is still the biggest provider and has retained its market share (56% in both 2011 and 2016), its LV and PCS systems which dominated the market in 2011 are hardly used anymore, with almost all practices having transitioned to the Web system. The use of TPP's SystmOne has increased from 18% to 34%, while that of Vision by In Practice System has halved (from 18% to 9%). Many providers that were present in 2011 have subsequently withdrawn from primary care, with the exception of Microtest's Evolution (transitioned from Practice Manager). If the current trend continues, English primary care will be completely dominated by EMIS Web and SystmOne in the next 5 to 10 years, and access to both of these systems would ensure almost complete coverage for England.

The trend for primary care convergence to two clinical systems has implications for the future of PCDs and the research findings based on them. CPRD and THIN will need to adapt very quickly and include EMIS and/or SystmOne practices in their processes. Given that the CPRD and THIN are the two most widely used primary care databases in clinical research, losing them altogether – as happened with the DIN-LINK database [20] – would be a severe setback for the research community. As of 20 July 2017, a PubMed search identified 1,782 published papers linked to the CPRD (886 in the last 5 years), 471 linked to THIN (303 in the last 5 years), 71 linked to QRESEARCH (32 in the last 5 years) and 2 to ResearchOne (both in the last 5 years).

For peer review only - http://bmjopen.bmj.com/site/about/guidelines.xhtml

Although not exhaustive, this search indicates the large variability across databases in terms of scientific contribution, demonstrating that the most accessible and productive databases are the ones at immediate risk.

Within the CPRD, there are clear actions towards future-proofing the resource, in light of the deterioration of the Vision market share. A large number of EMIS practices are already contributing data to the resource, but differences in the data format (compared to the standard Vision format) has prevented their immediate release along with Vision data, while it was not possible to link the EMIS data to other data sets. Nevertheless, a major transformation in processes is being undertaken which will allow the release of both Vision and EMIS data as standard, within 2018. In addition, the recruitment of EMIS practices continues, with over 150 practices having joined the CPRD in the last 12 months.

Users of the UK PCDs need to be aware of the generalisability issues we described, and consider if there are any risks relevant to their studies. Generalisability (external validity) should be discussed as standard in such work and is listed as an item (#21) in both the STROBE and RECORD statements.[21 22] The context is important here, and regional representativeness may be less relevant for clinical questions but more relevant for health services research. Sensitivity analyses on a more representative group of practices, obtained through deterministic sampling and existing software,[23] can also be used to strengthen findings.[24] However, the strong clustering of clinical systems within CCGs, largely driven top-down from CCGs to general practices, limits the usefulness of such sampling approaches.

Conclusions

The geographical representativeness of primary care databases varies enormously, and the two most used databases in the UK, the CPRD and THIN, were in 2016 the least representative of the major databases due to the quickly diminishing market share of the clinical computer system providing their data (Vision). The existence of these databases is under threat, and urgent action is required to allow data collection from at least one of the two dominant clinical systems (EMIS Web and SystmOne). CPRD has recognized this, and has recently negotiated access to data held by EMIS practices, and is due to operationalise this data by 2018. In addition, development and access barriers that have restricted publication outputs from data drawn from EMIS (QRESEARCH) and SystmOne (ResearchOne) practices urgently need to be overcome if the confidential use of NHS patient data is to continue driving research that directly informs patient safety, management, and health services policy.

For peer review only - http://bmjopen.bmj.com/site/about/guidelines.xhtml

Acknowledgments

We would like to thank the Office of National Statistics and NHS Digital for the wealth of information they have collected and systematically organised, which made this study possible.

Funding

MRC Health eResearch Centre Grant MR/K006665/1 supported the time and facilities of EK. The views expressed are those of the authors and not necessarily those of the MRC.

Declaration of competing interests

EK, RS, PH and DE are members of the Independent Scientific Advisory Committee (ISAC) for MHRA database research: https://www.gov.uk/government/groups/independent-scientific-advisory-committee-for-mhra-database-research

Ethical approval

Not applicable

Patient involvement

Not applicable

Authorship & contributions

EK designed the study, extracted the data from all sources, performed the analyses and drafted the first version of the manuscript. RS, PH, DE, TD and DA critically edited the manuscript. EK is the guarantor of this work and, as such, had full access to all the data in the study and takes responsibility for the integrity of the data and the accuracy of the data analysis.

Transparency declaration

EK affirms that this manuscript is an honest, accurate, and transparent account of the study being reported; that no important aspects of the study have been omitted; and that any discrepancies from the study as planned have been explained.

Data sharing

The data used in this study are freely available and the authors are happy to share an organised and cleaned final dataset.

For peer review only - http://bmjopen.bmj.com/site/about/guidelines.xhtml

References

1. Millman A, Lee N, Brooke A. ABC of medical computing. Computers in general practice--I. *Bmj* 1995;311:800-2.
2. Kontopantelis E, Buchan I, Reeves D, Checkland K, Doran T. Relationship between quality of care and choice of clinical computing system: retrospective analysis of family practice performance under the UK's quality and outcomes framework. *Bmj Open* 2013;3.
3. Roland M. Linking physicians' pay to the quality of care - A major experiment in the United Kingdom. *New Engl J Med* 2004;351:1448-54.
4. de Lusignan S, Chan T. The development of primary care information technology in the United kingdom. *J Ambul Care Manage* 2008;31:201-10.
5. NHS England. The Terminology Roadmap for the NHS: Recoomendations for the Standardisation Committee for Care Information. 2014:1-35. http://www.england.nhs.uk/iscg/wp-content/uploads/sites/4/2014/09/20140827_SCCI_Item_9_Report_v3.pdf
6. Tai TW, Anandarajah S, Dhoul N, de Lusignan S. Variation in clinical coding lists in UK general practice: a barrier to consistent data entry? *Inform Prim Care* 2007;15:143-50.
7. Springate DA, Kontopantelis E, Ashcroft DM, Olier I, Parisi R, Chamapiwa E, et al. ClinicalCodes: An Online Clinical Codes Repository to Improve the Validity and Reproducibility of Research Using Electronic Medical Records. *Plos One* 2014;9.
8. Vezyridis P, Timmons S. Evolution of primary care databases in UK: a scientometric analysis of research output. *Bmj Open* 2016;6.
9. Carbonari DM, Saine ME, Newcomb CW, Blak B, Roy JA, Haynes K, et al. Use of demographic and pharmacy data to identify patients included within both the Clinical Practice Research Datalink (CPRD) and The Health Improvement Network (THIN). *Pharmacoepidem Dr S* 2015;24:999-1003.
10. Hippisley-Cox J, Coupland C. Development and validation of risk prediction equations to estimate survival in patients with colorectal cancer: cohort study. *Bmj* 2017;357:j2497.
11. Doran T, Drever F, Whitehead M. Is there a north-south divide in social class inequalities in health in Great Britain? Cross sectional study using data from the 2001 census. *Brit Med J* 2004;328:1043-45.
12. Hacking JM, Muller S, Buchan IE. Trends in mortality from 1965 to 2008 across the English north-south divide: comparative observational study. *Brit Med J* 2011;342.
13. Buchan IE, Kontopantelis E, Sperrin M, Chandola T, Doran T. North-South disparities in English mortality1965-2015: longitudinal population study. *J Epidemiol Community Health* 2017;71:928-36.
14. Appleby J, Raleigh V, Frosini F, Bevan G, Gao H, Lyscom T. *Variations in health care: the good, the bad and the inexplicable*: King's Fund, 2011.
15. Bojke C, Castelli A, Street A, Ward P, Laudicella M. Regional Variation in the Productivity of the English National Health Service. *Health Econ* 2013;22:194-211.
16. NHS Digital. General and Personal Medical Services, England 2006-2016, as at 30 September, Experimental statistics. http://content.digital.nhs.uk/article/2021/Website-Search?productid=24768&q=+General+and+Personal+Medical+Services&sort=Relevance&size=10&page=1&area=both#top

For peer review only - http://bmjopen.bmj.com/site/about/guidelines.xhtml

17. Communities and Local Government. The English Indices of Deprivation 2015: Technical Report. Government DfCaL, 2015. https://www.gov.uk/government/publications/english-indices-of-deprivation-2015-technical-report
18. Office for National Statistics. Open Geography Portal. 2017. http://geoportal.statistics.gov.uk/
19. SPMAP: Stata module to visualize spatial data [program], 2017.
20. Carey IM, Cook DG, De Wilde S, Bremner SA, Richards N, Caine S, et al. Developing a large electronic primary care database (Doctors' Independent Network) for research. *Int J Med Inform* 2004;73:443-53.
21. von Elm E, Altman DG, Pocock SJ, Gotzsche PC, Vandenbroucke JP. Strengthening the reporting of observational studies in epidemiology (STROBE) statement: guidelines for reporting observational studies. *Brit Med J* 2007;335:806-08.
22. Benchimol EI, Smeeth L, Guttmann A, Harron K, Moher D, Petersen I, et al. The REporting of studies Conducted using Observational Routinely-collected health Data (RECORD) Statement. *Plos Med* 2015;12.
23. Kontopantelis E. A Greedy Algorithm for Representative Sampling: repsample in Stata. *J Stat Softw* 2013;55:1-19.
24. Kontopantelis E, Springate D, Reeves D, Ashcroft DM, Valderas JM, Doran T. Withdrawing performance indicators: retrospective analysis of general practice performance under UK Quality and Outcomes Framework. *Bmj-Brit Med J* 2014;348.

For peer review only

For peer review only - http://bmjopen.bmj.com/site/about/guidelines.xhtml

Table 1: Regional distributions of systems and the characteristics of their respective general practices*†‡§

	EMIS¶	SystmOne	Vision v3	Evolution
Aggregates (%)				
Number of practices	4199(56%)	2552(34%)	636(9%)	90(1%)
Number of patients	32191392(56%)	20199414(35%)	4601205(8%)	629166(1%)
Number of GPs	18675(57%)	11160(34%)	2433(7%)	393(1%)
Medians (25th and 75th centiles)				
IMD 2015#	22.2 (12.1,37.4)	22.5 (12.8,36.8)	22.4 (12.3,37.0)	22.7 (14.4,31.0)
List size	6833 (4257,10094)	7080 (4214,10553)	6279 (3988,9759)	6222 (4743,9121)
Patients aged 75 or over	476 (240,823)	524 (256,895)	455 (225,710)	592 (400,924)
Means (SD)				
All GPs	5.1(3.4)	5.1(3.7)	4.5(3.1)	5.5(2.9)
Female GPs	2.8(2.4)	2.7(2.5)	2.3(2.1)	2.7(2.1)
GPs aged under 40	1.7(1.9)	1.6(2.0)	1.4(1.7)	1.5(1.6)
GPs aged 40 to 54	2.3(1.9)	2.4(2.1)	2.0(1.8)	2.8(1.9)
GPs aged 55 or over	1.0(1.0)	1.0(1.0)	1.1(1.0)	1.2(1.1)
All Nurses	3.1(2.3)	3.6(2.6)	2.8(1.9)	3.4(1.6)
Regional counts, NHS regions (%)				
Wessex	164(55%)	113(38%)	17(6%)	4(1%)
London	917(68%)	254(19%)	182(13%)	1(0%)
Yorkshire & the Humber	186(25%)	544(74%)	5(1%)	0(0%)
Cumbria & the North East	270(59%)	172(38%)	12(3%)	0(0%)
Cheshire & Merseyside	353(92%)	19(5%)	8(2%)	2(1%)
North Midlands	260(54%)	216(45%)	2(0%)	2(0%)
West Midlands	496(76%)	96(15%)	58(9%)	0(0%)
Central Midlands	156(28%)	378(69%)	16(3%)	0(0%)
East	112(21%)	413(77%)	4(1%)	4(1%)
South West	225(59%)	86(22%)	7(2%)	65(17%)
South East	303(56%)	96(18%)	145(27%)	1(0%)
South Central	227(55%)	129(31%)	57(14%)	3(1%)
Greater Manchester	310(65%)	36(8%)	123(26%)	8(2%)
Lancashire	220(100%)	0(0%)	0(0%)	0(0%)

* Data for Aug 2016 (clinical system) and Sep 2016 (GMS data)
† System information not available for 49 (0.65%) of 7,526 practices
‡ All GP numbers exclude locums
§ SystmOne provided by TPP, Vision (version 3) provided by In Practice Systems, Evolution provided by Microtest
¶ EMIS includes Web (4,176 practices), LV (19 practices) and PCS (4 practices)
# Index of Multiple Deprivation, details available in the 2015 technical report of the English Indices of Deprivation[17]

For peer review only
For peer review only - http://bmjopen.bmj.com/site/about/guidelines.xhtml

Figure 1: Spatial map at the Clinical Commissioning Group (CCG) level, September 2016: EMIS*†

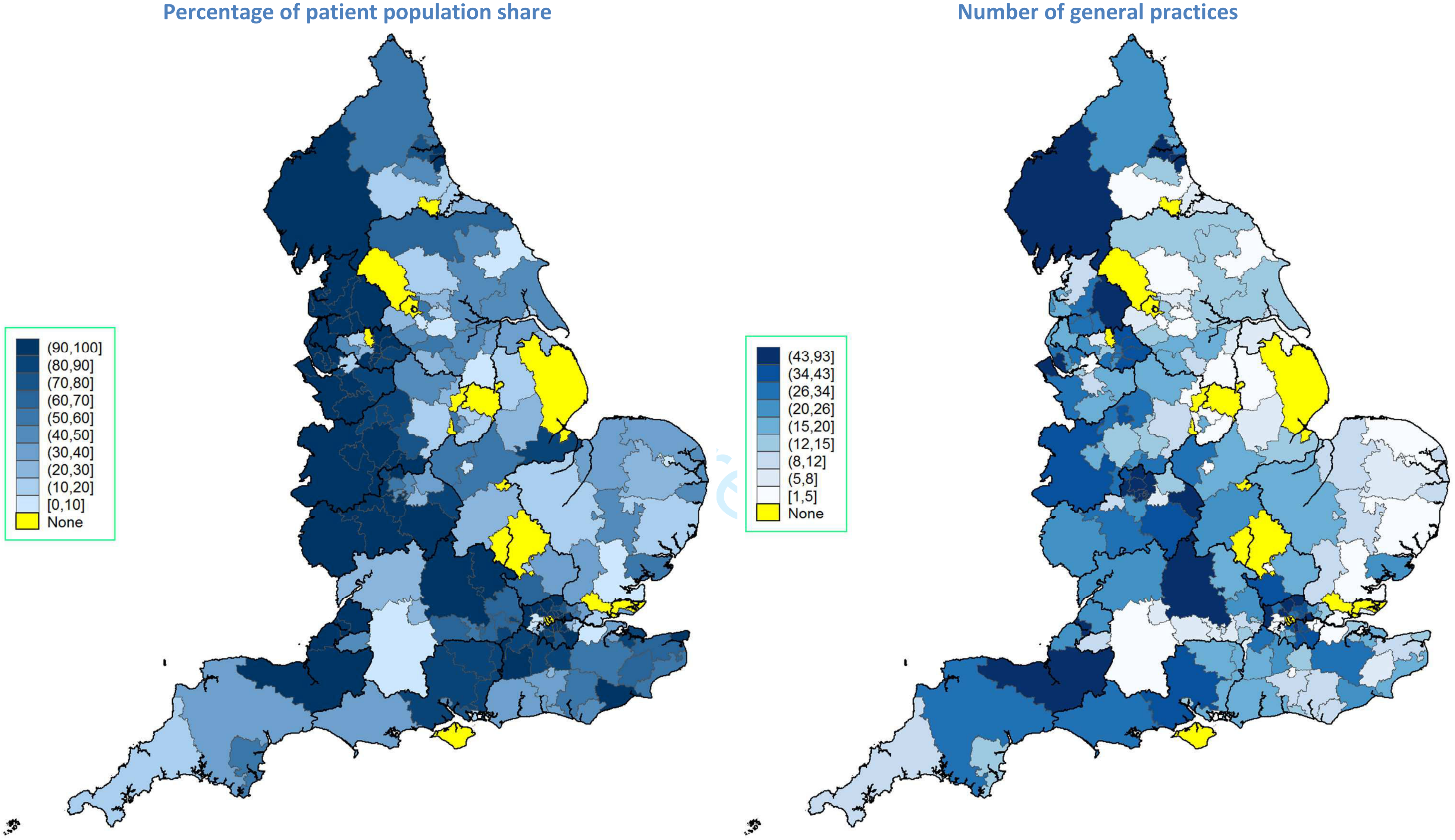

* thicker border lines correspond to the 14 NHS regions

† left graph uses equidistant class breaks; right graph uses class breaks based on distribution of variable of interest, with each class having approximately the same number of spatial polygons (CCGs)

For peer review only - http://bmjopen.bmj.com/site/about/guidelines.xhtml

Figure 2: Spatial map at the Clinical Commissioning Group (CCG) level, September 2016: SystmOne*†

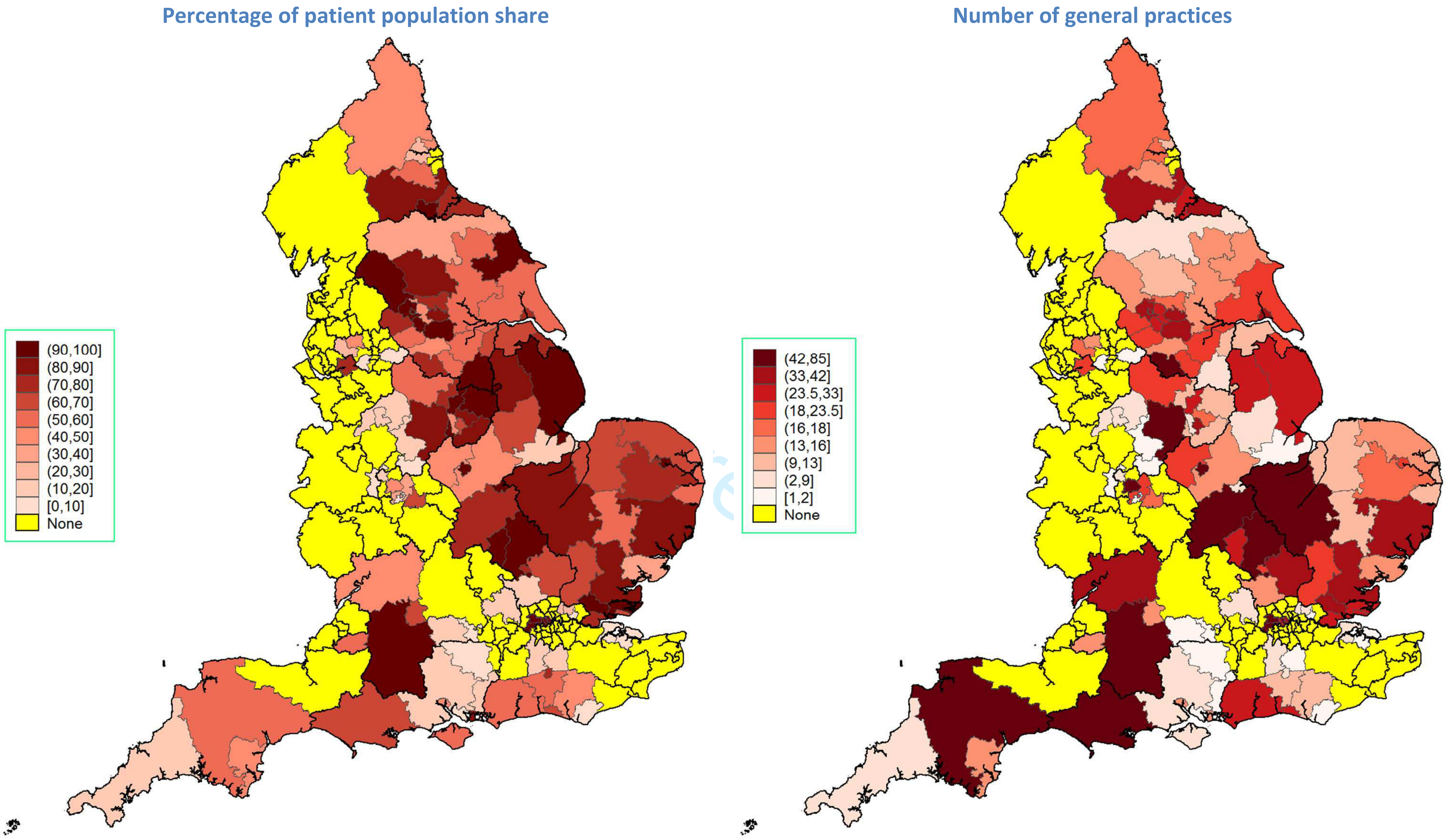

* thicker border lines correspond to the 14 NHS regions
† left graph uses equidistant class breaks; right graph uses class breaks based on distribution of variable of interest, with each class having approximately the same number of spatial polygons (CCGs)

For peer review only - http://bmjopen.bmj.com/site/about/guidelines.xhtml

Figure 3: Spatial map at the Clinical Commissioning Group (CCG) level, September 2016: Vision*†

Percentage of patient population share | Number of general practices

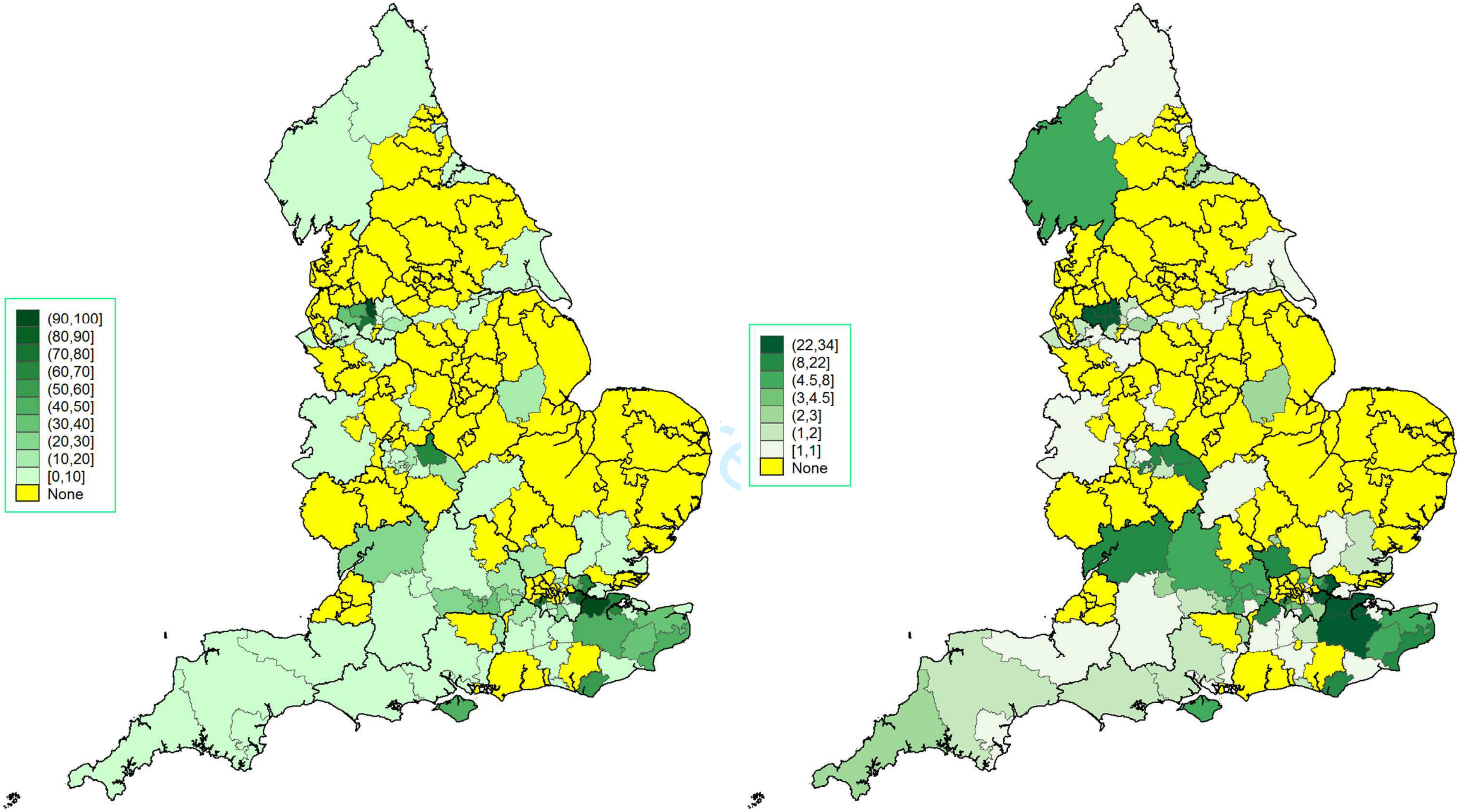

* thicker border lines correspond to the 14 NHS regions

† left graph uses equidistant class breaks; right graph uses class breaks based on distribution of variable of interest, with each class having approximately the same number of spatial polygons (CCGs)

For peer review only - http://bmjopen.bmj.com/site/about/guidelines.xhtml

STROBE 2007 (v4) Statement—Checklist of items that should be included in reports of *cross-sectional studies*

Section/Topic	Item #	Recommendation	Reported on page #
**Title and abstract**	1	(*a*) Indicate the study's design with a commonly used term in the title or the abstract	2
		(*b*) Provide in the abstract an informative and balanced summary of what was done and what was found	2
**Introduction**			
Background/rationale	2	Explain the scientific background and rationale for the investigation being reported	4-5
Objectives	3	State specific objectives, including any prespecified hypotheses	5
**Methods**			
Study design	4	Present key elements of study design early in the paper	5-6
Setting	5	Describe the setting, locations, and relevant dates, including periods of recruitment, exposure, follow-up, and data collection	5-6
Participants	6	(*a*) Give the eligibility criteria, and the sources and methods of selection of participants	5-6
Variables	7	Clearly define all outcomes, exposures, predictors, potential confounders, and effect modifiers. Give diagnostic criteria, if applicable	5-6
Data sources/ measurement	8*	For each variable of interest, give sources of data and details of methods of assessment (measurement). Describe comparability of assessment methods if there is more than one group	5-6
Bias	9	Describe any efforts to address potential sources of bias	NA
Study size	10	Explain how the study size was arrived at	5-6
Quantitative variables	11	Explain how quantitative variables were handled in the analyses. If applicable, describe which groupings were chosen and why	6
Statistical methods	12	(*a*) Describe all statistical methods, including those used to control for confounding	NA
		(*b*) Describe any methods used to examine subgroups and interactions	NA
		(*c*) Explain how missing data were addressed	NA
		(*d*) If applicable, describe analytical methods taking account of sampling strategy	NA
		(*e*) Describe any sensitivity analyses	NA
**Results**			

For peer review only

Participants	13*	(a) Report numbers of individuals at each stage of study—eg numbers potentially eligible, examined for eligibility, confirmed eligible, included in the study, completing follow-up, and analysed	6
		(b) Give reasons for non-participation at each stage	NA
		(c) Consider use of a flow diagram	NA
Descriptive data	14*	(a) Give characteristics of study participants (eg demographic, clinical, social) and information on exposures and potential confounders	6 and 12
		(b) Indicate number of participants with missing data for each variable of interest	6
Outcome data	15*	Report numbers of outcome events or summary measures	6 and 12
Main results	16	(*a*) Give unadjusted estimates and, if applicable, confounder-adjusted estimates and their precision (eg, 95% confidence interval). Make clear which confounders were adjusted for and why they were included	NA
		(*b*) Report category boundaries when continuous variables were categorized	12
		(*c*) If relevant, consider translating estimates of relative risk into absolute risk for a meaningful time period	NA
Other analyses	17	Report other analyses done—eg analyses of subgroups and interactions, and sensitivity analyses	NA
**Discussion**			
Key results	18	Summarise key results with reference to study objectives	6-7
Limitations	19	Discuss limitations of the study, taking into account sources of potential bias or imprecision. Discuss both direction and magnitude of any potential bias	7
Interpretation	20	Give a cautious overall interpretation of results considering objectives, limitations, multiplicity of analyses, results from similar studies, and other relevant evidence	7-8
Generalisability	21	Discuss the generalisability (external validity) of the study results	7-8
**Other information**			
Funding	22	Give the source of funding and the role of the funders for the present study and, if applicable, for the original study on which the present article is based	9

*Give information separately for cases and controls in case-control studies and, if applicable, for exposed and unexposed groups in cohort and cross-sectional studies.

Note: An Explanation and Elaboration article discusses each checklist item and gives methodological background and published examples of transparent reporting. The STROBE checklist is best used in conjunction with this article (freely available on the Web sites of PLoS Medicine at http://www.plosmedicine.org/, Annals of Internal Medicine at http://www.annals.org/, and Epidemiology at http://www.epidem.com/). Information on the STROBE Initiative is available at www.strobe-statement.org.

For peer review only

BMJ Open

Spatial distribution of clinical computer systems in primary care in England in 2016 and implications for primary care electronic medical record databases: a cross sectional population study

Journal:	*BMJ Open*
Manuscript ID	bmjopen-2017-020738.R1
Article Type:	Research
Date Submitted by the Author:	30-Nov-2017
Complete List of Authors:	Kontopantelis, Evangelos; University of Manchester, Division of Informatics, Imaging and Data Sciences; Faculty of Biology, Medicine and Health; NIHR School for Primary Care Research, Centre for Primary Care<br>Stevens, Richard; University of Oxford, Nuffield Dept Primary Care Health Sciences<br>Helms, Peter; University of Aberdeen, The Institute of Applied Health Sciences<br>Edwards, Duncan; University of Cambridge, Department of Public Health and Primary Care<br>Doran, Tim; University of York, Department of Health Sciences<br>Ashcroft, Darren; University of Manchester, Division of Pharmacy & Optometry; Faculty of Biology, Medicine and Health
**Primary Subject Heading**:	General practice / Family practice
Secondary Subject Heading:	Epidemiology, Health informatics, Health services research, Research methods
Keywords:	Electronic Health Records, Primary Care Databases, clinical computer systems, EMIS, Vision, CPRD

SCHOLARONE™
Manuscripts

Spatial distribution of clinical computer systems in primary care in England in 2016 and implications for primary care electronic medical record databases: a cross sectional population study

Evangelos Kontopantelis[1 2], Richard Stevens[3], Peter Helms[4], Duncan Edwards[5], Tim Doran[6], Darren M Ashcroft[1 7].

[1] NIHR School for Primary Care Research; University of Manchester; Manchester, UK.
[2] Division of Informatics, Imaging and Data Sciences; Faculty of Biology, Medicine and Health; University of Manchester; Manchester, UK.
[3] NIHR School for Primary Care Research; Nuffield Department of Health Care Sciences; University of Oxford, Oxford, UK.
[4] The Institute of Applied Health Sciences; University of Aberdeen.
[5] Department of Public Health and Primary Care; School of Clinical Medicine; University of Cambridge; Cambridge, UK.
[6] Department of Health Sciences; University of York; York, UK.
[7] Division of Pharmacy & Optometry; Faculty of Biology, Medicine and Health; University of Manchester; Manchester, UK.

Correspondence to:
Evangelos Kontopantelis
Division of Informatics, Imaging and Data Sciences, University of Manchester
5th floor Williamson Building
University of Manchester
e-mail: e.kontopantelis@manchester.ac.uk

Keywords

For peer review only

Abstract

Objectives

UK Primary Care Databases (PCDs) are used by researchers worldwide to inform clinical practice. These databases have been primarily tied to single clinical computer systems, but little is known about the adoption of these systems by primary care practices or their geographical representativeness. We explore the spatial distribution of clinical computing systems and discuss the implications for the longevity and regional representativeness of these resources.

Design

Cross-sectional study.

Setting

English primary care clinical computer systems.

Participants

7,526 general practices in August 2016.

Methods

Spatial mapping of family practices in England in 2016 by clinical computer system at two geographical levels, the lower Clinical Commissioning Group (CCG, 209 units) and the higher NHS regions (14 units). Data for practices included, numbers of doctors, nurses, and patients; and area deprivation.

Results

Of 7,526 practices, EMIS was used in 4,199 (56%), SystmOne in 2,552 (34%) and Vision in 636 (9%). Great regional variability was observed for all systems, with EMIS having a stronger presence in the West of England, London and the South; SystmOne in the East and some regions in the South; and Vision in London, the South, Greater Manchester and Birmingham.

Conclusions

PCDs based on single clinical computer systems are geographically clustered in England. For example, CPRD and THIN, the most popular primary care databases in terms of research outputs, are based on the Vision clinical computer system, used by less than 10% of practices and heavily concentrated in three major conurbations and the South. Researchers need to be aware of the analytical challenges posed by clustering, and barriers to accessing alternative PCDs need to be removed.

Keywords

Electronic Health Records; Primary Care Databases; clinical computer systems; representativeness; EMIS; Vision; SystmOne; CPRD; THIN; QRESEARCH; ResearchOne.

For peer review only - http://bmjopen.bmj.com/site/about/guidelines.xhtml

Strengths and limitations of the study

- Cross-sectional analysis of all clinical computer systems used in English primary care, in August 2016.
- Data allowed a detailed description of regional use of each clinical computer system at the Clinical Commissioning Group (CCG) level, and the discussion of implications for UK Primary Care Databases (PCDs).
- Although regional presence of a clinical computer system cannot be equated to contribution to a PCD, since contributing practices are anonymised, inferences on the regional representativeness of UK PCDs are still possible.

For peer review only

For peer review only - http://bmjopen.bmj.com/site/about/guidelines.xhtml

Introduction

Primary care in the UK has been almost fully computerised since the early 1990s. [1] Following the introduction of partial subsidies for the purchase of clinical computer systems in 1998 and full subsidies in 2003 (in anticipation of the implementation of a national pay-for-performance programme) UK primary care became fully computerised.[2 3] Interoperability requirements of the National Health Service led to the universal adoption of a loosely hierarchical clinical coding system, known as Read codes,[4] which is due to be replaced in April 2018 by a multi-hierarchical coding system (SNOMED CT).[5] Various commercial providers were permitted to enter the market for clinical computer systems, resulting in numerous different systems with varying interfaces, mechanisms and implementations of Read code usage.[6] By 2010-11, seven clinical computer systems were consistently active in England, holding 99% of the market share: EMIS systems (LV and PC) were active in 54.7% of practices, followed by Vision v3 (18.1%) and SystmOne (17.8%), with the remaining 9.4% held by other systems (Synergy, Practice Manager, Premiere and the then newly launched EMIS Web).[2]

The uniformity and interoperability standards have facilitated the creation of large repositories of primary care electronic health records (EHRs), which contain the complete primary care records of patients attending general practices in the UK. The secondary use of these EHRs by researchers – both within and outside the UK – has been increasing exponentially,[7] and they have provided insights in numerous research areas, including real-world effectiveness, adverse events, resource utilisation, condition prevalence and incidence, quality of care, and policy interventions.[8 9] Several EHR databases exist, maintained by the different clinical computer system providers, drawing data from practices using their systems that have agreed to make patient data available for secondary use. The four largest EHR databases (hereafter primary care databases, or PCDs) in terms of numbers of patient records are the Clinical Practice Research Datalink (CPRD), The Health Improvement Network (THIN), QResearch and ResearchOne.

The CPRD (formally General Practice Research Database, GPRD) was established in 1987 and has been owned by the Secretary of State for Health since 1994. In May 2017, the CPRD covered approximately 8% of the UK population, with 718 contributing general practices and over 17 million total patients (historical and current). The CPRD primarily collects data from Vision practices, although it is currently undergoing an expansion to include EMIS practices, and a future expansion to cover SystmOne practices is planned. THIN was established in 2003 as a collaboration between the company owning Vision (In Practice Systems Ltd) and the CSD Medical Research Group (now Quintiles IMS). In April 2015, THIN reported covering 6% of the UK population, with 562 practices and 11 million total patients. There is a considerable overlap (around 60%) between CPRD and THIN practices, which has implications for studies wishing to

For peer review only - http://bmjopen.bmj.com/site/about/guidelines.xhtml

replicate findings between different databases.[10] QResearch collects data from practices using EMIS systems and is the biggest PCD, with approximately 1500 practices in 2017, covering a population of more than 22 million patients.[11] ResearchOne is a collaboration between the provider of SystmOne (TPP) and the University of Leeds, reporting 28 million (primary and secondary care) records and 423 practices in 2017.

The geographical coverage of PCDs is dependent on the location of practices using the parent clinical computer system, which is in turn dependent on historical patterns of market penetration by the software suppliers and system uptake by general practices. Geographical representativeness is an important prerequisite if analysts are to generalise PCD findings to the whole of England and the UK, which is what routinely happens in practice. This is due to great regional variability across England in terms of population characteristics (primarily: age, ethnicity and deprivation),[12] or even regional variation in hard outcomes. For example, a persistent mortality divide between North and the South of England has existed since the middle of the previous century,[13] while, more recently, much higher mortality rates were observed for young adults in the North of England.[14] There is also regional variation in the organisation and productivity of health services in England,[15 16] which could have important implications for the generalisability of health services research with the use of regionally unrepresentative PCDs. The aim of this paper is to describe the regional distribution of clinical computer systems in English primary care, evaluate the implications of the current picture of representativeness and provide some insight into the sustainability of existing PCDs.

Methods

Data

Clinical computer system information was obtained from NHS Digital after direct communication, for August 2016. Primary care workforce and patient information as of 30 September 2016 was downloaded from the NHS Digital website.[17] At the practice level, information was available on geography (Clinical Commissioning Group and NHS region), patient list size by age groups, and numbers and full-time equivalent (FTE) for GPs and nurses. Deprivation was quantified using the 2015 release of the Index of Multiple Deprivation (IMD), a complete aggregate measure widely used to quantify area deprivation, attributed to the practice location.[18] Spatial coordinates for NHS organisational units in 2016 were obtained from the ONS open geography portal.[19] We focused on two organisational levels, the lower Clinical Commissioning Groups (CCGs) with 209 units, and the higher NHS regions with 14 units.

For peer review only - http://bmjopen.bmj.com/site/about/guidelines.xhtml

Analyses

For all aspects of data manipulation and analysis we used Stata v14.1. Whenever medians are reported, we also report the 25th and 75th centiles. Spatial maps were plotted using the *spmap* command.[20] Practice-level data were aggregated by clinical computer system, to provide information on all patients, patients aged 75 or over, GPs and nurses, practice location deprivation and list size. Counts for each clinical computer system, by NHS region, were also calculated. Spatial graphs at the CCG level, with additional information on NHS regions, were plotted for the three most popular clinical computer systems, to provide a visual guide in regional distribution and representativeness.

Results

System information was missing for 49 (0.7%) of 7,526 general practices. EMIS systems were used in 4,199 practices (56%), with all but 23 of these using EMIS Web. SystmOne was used in 2,552 (34%), Vision in 636 (9%) and Evolution in 90 (1%) practices. Patterns of area deprivation, based on the locations of general practices, were similar across all systems. SystmOne practices tended to be larger (median of 7,080 patients), followed by EMIS (6,833), Vision (6,279) and Evolution (6,222).

Great regional variability in system usage was observed both at the NHS Region level (Table 1) and CCG level (Figures 1-3). EMIS is present in all but 18 of the 209 CCGs (91.4%), with a much stronger presence in the West of England, London and the South. SystmOne is present in 120 CCGs (57.4%), and is mainly active in the East and some regions in the South. Vision, although with a much lower market share than SystmOne, is still used in 96 CCGs (45.9%), mainly in London, the South, Greater Manchester and Birmingham. Evolution is only present in 18 CCGs (8.6%) and is primarily used in the South West.

<Please place Table 1 here>

<Please place Figure 1 here>

<Please place Figure 2 here>

<Please place Figure 3 here>

Discussion

High regional variability exists in the use of different clinical computer systems in English primary care, which should be a consideration when utilising primary care electronic health databases based on this population in the future, especially if effect heterogeneity (or other forms of heterogeneity) is context relevant. For example, drawing nationwide conclusions in health services organisation would be more problematic than identifying medication side-

For peer review only - http://bmjopen.bmj.com/site/about/guidelines.xhtml

effects. EMIS Web is by far the most widely used clinical computer system and therefore QResearch is the most nationally representative single database – potentially able to collect data from almost all English CCGs. SystmOne has a very strong presence in many parts of England, but no presence in many CCGs in the North West, West Midlands, London and South East. The ResearchOne database is therefore unable to capture data from many regions. Finally, Vision is the most geographically restricted of the three major clinical computer systems, with relatively few practices heavily concentrated in three conurbations and the South. The CPRD and THIN databases are therefore currently unable to provide comprehensive coverage of large parts of the country, particularly in the North and East of England.

Strengths and limitations of the study

The main strength of this study is the use of numerous national administrative datasets of high data quality, allowing us to obtain a complete picture for the whole of England. The main weakness of the work is the fact we cannot equate the regional presence of a clinical system to active contribution to a primary care database – not all practices contribute data and contributing practices are anonymised – and we have therefore discussed potential contribution instead. Additional information on currently registered patients would have been relevant, but is not routinely available for non-users of the resources (but can be deduced by users).

Findings and implications

The current picture of clinical system usage in English primary care is very different to what was reported for 2011.[2] Although EMIS is still the biggest provider and has retained its market share (56% in both 2011 and 2016), its LV and PCS systems which dominated the market in 2011 are hardly used anymore, with almost all practices having transitioned to the Web system. The use of TPP's SystmOne has increased from 18% to 34%, while that of Vision by In Practice System has halved (from 18% to 9%). Many providers that were present in 2011 have subsequently withdrawn from primary care, with the exception of Microtest's Evolution (transitioned from Practice Manager). If the current trend continues, English primary care will be completely dominated by EMIS Web and SystmOne in the next 5 to 10 years, and access to both of these systems would ensure almost complete coverage for England.

The trend for primary care convergence to two clinical systems has implications for the future of PCDs and the research findings based on them. CPRD and THIN will need to adapt very quickly and include EMIS and/or SystmOne practices in their processes. Given that the CPRD and THIN are the two most widely used primary care databases in clinical research, losing them altogether – as happened with the DIN-LINK database [21] – would be a severe setback for the research community. As of 20 July 2017, a PubMed search identified 1,782 published papers

For peer review only - http://bmjopen.bmj.com/site/about/guidelines.xhtml

linked to the CPRD (886 in the last 5 years), 471 linked to THIN (303 in the last 5 years), 71 linked to QRESEARCH (32 in the last 5 years) and 2 to ResearchOne (both in the last 5 years). Although not exhaustive, this search indicates the large variability across databases in terms of scientific contribution, demonstrating that the most accessible and productive databases are the ones at immediate risk.

Within the CPRD, there are clear actions towards future-proofing the resource, in light of the deterioration of the Vision market share. A large number of EMIS practices are already contributing data to the resource, but differences in the data format (compared to the standard Vision format) has prevented their immediate release along with Vision data, while it was not possible to link the EMIS data to other data sets. Nevertheless, a major transformation in processes is being undertaken which will allow the release of both Vision and EMIS data as standard, within 2018. In addition, the recruitment of EMIS practices continues, with over 150 practices having joined the CPRD in the last 12 months.

Users of the UK PCDs need to be aware of the generalisability issues we described, and consider if there are any risks relevant to their studies. Generalisability (external validity) should be discussed as standard in such work and is listed as an item (#21) in both the STROBE and RECORD statements.[22 23] The context is important here, and regional representativeness may be less relevant for clinical questions but more relevant for health services research. Sensitivity analyses on a more representative group of practices, obtained through deterministic sampling and existing software,[24] can also be used to strengthen findings.[25] However, the strong clustering of clinical systems within CCGs, largely driven top-down from CCGs to general practices, limits the usefulness of such sampling approaches.

Conclusions

The geographical representativeness of primary care databases varies enormously, and the two most used databases in the UK, the CPRD and THIN, were in 2016 the least representative of the major databases due to the quickly diminishing market share of the clinical computer system providing their data (Vision). The existence of these databases is under threat, and urgent action is required to allow data collection from at least one of the two dominant clinical systems (EMIS Web and SystmOne). CPRD has recognized this, and has recently negotiated access to data held by EMIS practices, and is due to operationalise this data by 2018. In addition, development and access barriers that have restricted publication outputs from data drawn from EMIS (QRESEARCH) and SystmOne (ResearchOne) practices urgently need to be overcome if the confidential use of NHS patient data is to continue driving research that directly informs patient safety, management, and health services policy.

For peer review only - http://bmjopen.bmj.com/site/about/guidelines.xhtml

For peer review only

For peer review only - http://bmjopen.bmj.com/site/about/guidelines.xhtml

Acknowledgments

We would like to thank the Office of National Statistics and NHS Digital for the wealth of information they have collected and systematically organised, which made this study possible.

Funding

MRC Health eResearch Centre Grant MR/K006665/1 supported the time and facilities of EK. The views expressed are those of the authors and not necessarily those of the MRC.

Declaration of competing interests

EK, RS, PH and DE are members of the Independent Scientific Advisory Committee (ISAC) for MHRA database research: https://www.gov.uk/government/groups/independent-scientific-advisory-committee-for-mhra-database-research

Ethical approval

Not applicable

Patient involvement

Not applicable

Authorship & contributions

EK designed the study, extracted the data from all sources, performed the analyses and drafted the first version of the manuscript. RS, PH, DE, TD and DA critically edited the manuscript. EK is the guarantor of this work and, as such, had full access to all the data in the study and takes responsibility for the integrity of the data and the accuracy of the data analysis.

Transparency declaration

EK affirms that this manuscript is an honest, accurate, and transparent account of the study being reported; that no important aspects of the study have been omitted; and that any discrepancies from the study as planned have been explained.

Data sharing

The data used in this study are freely available and the authors are happy to share an organised and cleaned final dataset.

For peer review only - http://bmjopen.bmj.com/site/about/guidelines.xhtml

References

1. Millman A, Lee N, Brooke A. ABC of medical computing. Computers in general practice--I. *Bmj* 1995;311:800-2.
2. Kontopantelis E, Buchan I, Reeves D, Checkland K, Doran T. Relationship between quality of care and choice of clinical computing system: retrospective analysis of family practice performance under the UK's quality and outcomes framework. *Bmj Open* 2013;3.
3. Roland M. Linking physicians' pay to the quality of care - A major experiment in the United Kingdom. *New Engl J Med* 2004;351:1448-54.
4. de Lusignan S, Chan T. The development of primary care information technology in the United kingdom. *J Ambul Care Manage* 2008;31:201-10.
5. NHS England. The Terminology Roadmap for the NHS: Recoomendations for the Standardisation Committee for Care Information. 2014:1-35. http://www.england.nhs.uk/iscg/wp-content/uploads/sites/4/2014/09/20140827_SCCI_Item_9_Report_v3.pdf
6. Tai TW, Anandarajah S, Dhoul N, de Lusignan S. Variation in clinical coding lists in UK general practice: a barrier to consistent data entry? *Inform Prim Care* 2007;15:143-50.
7. Springate DA, Kontopantelis E, Ashcroft DM, Olier I, Parisi R, Chamapiwa E, et al. ClinicalCodes: An Online Clinical Codes Repository to Improve the Validity and Reproducibility of Research Using Electronic Medical Records. *Plos One* 2014;9.
8. Thiru K, Hassey A, Sullivan F. Systematic review of scope and quality of electronic patient record data in primary care. *Bmj* 2003;326:1070.
9. Vezyridis P, Timmons S. Evolution of primary care databases in UK: a scientometric analysis of research output. *Bmj Open* 2016;6.
10. Carbonari DM, Saine ME, Newcomb CW, Blak B, Roy JA, Haynes K, et al. Use of demographic and pharmacy data to identify patients included within both the Clinical Practice Research Datalink (CPRD) and The Health Improvement Network (THIN). *Pharmacoepidem Dr S* 2015;24:999-1003.
11. Hippisley-Cox J, Coupland C. Development and validation of risk prediction equations to estimate survival in patients with colorectal cancer: cohort study. *Bmj* 2017;357:j2497.
12. Doran T, Drever F, Whitehead M. Is there a north-south divide in social class inequalities in health in Great Britain? Cross sectional study using data from the 2001 census. *Brit Med J* 2004;328:1043-45.
13. Hacking JM, Muller S, Buchan IE. Trends in mortality from 1965 to 2008 across the English north-south divide: comparative observational study. *Brit Med J* 2011;342.
14. Buchan IE, Kontopantelis E, Sperrin M, Chandola T, Doran T. North-South disparities in English mortality1965-2015: longitudinal population study. *J Epidemiol Community Health* 2017;71:928-36.
15. Appleby J, Raleigh V, Frosini F, Bevan G, Gao H, Lyscom T. *Variations in health care: the good, the bad and the inexplicable*: King's Fund, 2011.
16. Bojke C, Castelli A, Street A, Ward P, Laudicella M. Regional Variation in the Productivity of the English National Health Service. *Health Econ* 2013;22:194-211.
17. NHS Digital. General and Personal Medical Services, England 2006-2016, as at 30 September, Experimental statistics. http://content.digital.nhs.uk/article/2021/Website-

For peer review only - http://bmjopen.bmj.com/site/about/guidelines.xhtml

Search?productid=24768&q=+General+and+Personal+Medical+Services&sort=Relevance&size=10&page=1&area=both#top
18. Communities and Local Government. The English Indices of Deprivation 2015: Technical Report. Government DfCaL, 2015. https://www.gov.uk/government/publications/english-indices-of-deprivation-2015-technical-report
19. Office for National Statistics. Open Geography Portal. 2017. http://geoportal.statistics.gov.uk/
20. SPMAP: Stata module to visualize spatial data [program], 2017.
21. Carey IM, Cook DG, De Wilde S, Bremner SA, Richards N, Caine S, et al. Developing a large electronic primary care database (Doctors' Independent Network) for research. *Int J Med Inform* 2004;73:443-53.
22. von Elm E, Altman DG, Pocock SJ, Gotzsche PC, Vandenbroucke JP. Strengthening the reporting of observational studies in epidemiology (STROBE) statement: guidelines for reporting observational studies. *Brit Med J* 2007;335:806-08.
23. Benchimol EI, Smeeth L, Guttmann A, Harron K, Moher D, Petersen I, et al. The REporting of studies Conducted using Observational Routinely-collected health Data (RECORD) Statement. *Plos Med* 2015;12.
24. Kontopantelis E. A Greedy Algorithm for Representative Sampling: repsample in Stata. *J Stat Softw* 2013;55:1-19.
25. Kontopantelis E, Springate D, Reeves D, Ashcroft DM, Valderas JM, Doran T. Withdrawing performance indicators: retrospective analysis of general practice performance under UK Quality and Outcomes Framework. *Bmj-Brit Med J* 2014;348.

For peer review only

For peer review only - http://bmjopen.bmj.com/site/about/guidelines.xhtml

Table 1: Regional distributions of systems and the characteristics of their respective general practices*†‡§

	EMIS¶	SystmOne	Vision v3	Evolution
Aggregates (%)				
Number of practices	4199(56%)	2552(34%)	636(9%)	90(1%)
Number of patients	32191392(56%)	20199414(35%)	4601205(8%)	629166(1%)
Number of GPs	18675(57%)	11160(34%)	2433(7%)	393(1%)
Medians (25th and 75th centiles)				
IMD 2015#	22.2 (12.1,37.4)	22.5 (12.8,36.8)	22.4 (12.3,37.0)	22.7 (14.4,31.0)
List size	6833 (4257,10094)	7080 (4214,10553)	6279 (3988,9759)	6222 (4743,9121)
Patients aged 75 or over	476 (240,823)	524 (256,895)	455 (225,710)	592 (400,924)
Means (SD)				
All GPs	5.1(3.4)	5.1(3.7)	4.5(3.1)	5.5(2.9)
Female GPs	2.8(2.4)	2.7(2.5)	2.3(2.1)	2.7(2.1)
GPs aged under 40	1.7(1.9)	1.6(2.0)	1.4(1.7)	1.5(1.6)
GPs aged 40 to 54	2.3(1.9)	2.4(2.1)	2.0(1.8)	2.8(1.9)
GPs aged 55 or over	1.0(1.0)	1.0(1.0)	1.1(1.0)	1.2(1.1)
All Nurses	3.1(2.3)	3.6(2.6)	2.8(1.9)	3.4(1.6)
Regional counts, NHS regions (%)				
Wessex	164(55%)	113(38%)	17(6%)	4(1%)
London	917(68%)	254(19%)	182(13%)	1(0%)
Yorkshire & the Humber	186(25%)	544(74%)	5(1%)	0(0%)
Cumbria & the North East	270(59%)	172(38%)	12(3%)	0(0%)
Cheshire & Merseyside	353(92%)	19(5%)	8(2%)	2(1%)
North Midlands	260(54%)	216(45%)	2(0%)	2(0%)
West Midlands	496(76%)	96(15%)	58(9%)	0(0%)
Central Midlands	156(28%)	378(69%)	16(3%)	0(0%)
East	112(21%)	413(77%)	4(1%)	4(1%)
South West	225(59%)	86(22%)	7(2%)	65(17%)
South East	303(56%)	96(18%)	145(27%)	1(0%)
South Central	227(55%)	129(31%)	57(14%)	3(1%)
Greater Manchester	310(65%)	36(8%)	123(26%)	8(2%)
Lancashire	220(100%)	0(0%)	0(0%)	0(0%)

* Data for Aug 2016 (clinical system) and Sep 2016 (GMS data)
† System information not available for 49 (0.65%) of 7,526 practices
‡ All GP numbers exclude locums
§ SystmOne provided by TPP, Vision (version 3) provided by In Practice Systems, Evolution provided by Microtest
¶ EMIS includes Web (4,176 practices), LV (19 practices) and PCS (4 practices)
# Index of Multiple Deprivation (higher score implies higher levels of deprivation); details available in the 2015 technical report of the English Indices of Deprivation[18]

For peer review only - http://bmjopen.bmj.com/site/about/guidelines.xhtml

Figure 1: Spatial map at the Clinical Commissioning Group (CCG) level, September 2016: EMIS*†

* thicker border lines correspond to the 14 NHS regions
† left graph uses equidistant class breaks; right graph uses class breaks based on distribution of variable of interest, with each class having approximately the same number of spatial polygons (CCGs)

Figure 2: Spatial map at the Clinical Commissioning Group (CCG) level, September 2016: SystmOne*†

* thicker border lines correspond to the 14 NHS regions
† left graph uses equidistant class breaks; right graph uses class breaks based on distribution of variable of interest, with each class having approximately the same number of spatial polygons (CCGs)

Figure 3: Spatial map at the Clinical Commissioning Group (CCG) level, September 2016: Vision*†

* thicker border lines correspond to the 14 NHS regions
† left graph uses equidistant class breaks; right graph uses class breaks based on distribution of variable of interest, with each class having approximately the same number of spatial polygons (CCGs)

For peer review only

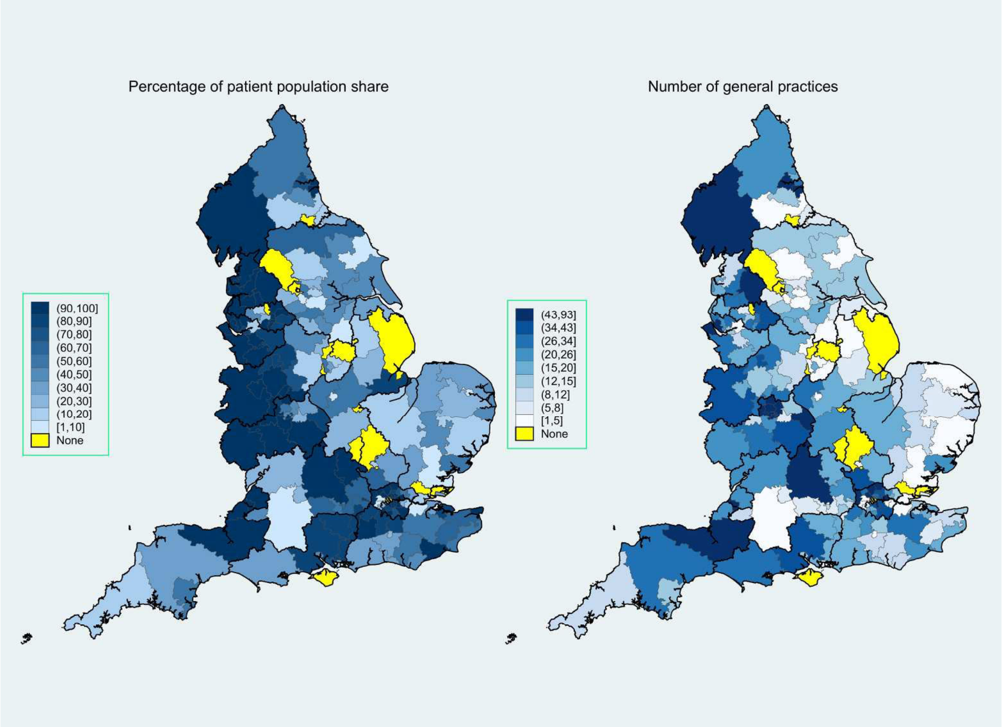

Figure 1: Spatial map at the Clinical Commissioning Group (CCG) level, September 2016: EMIS

101x73mm (600 x 600 DPI)

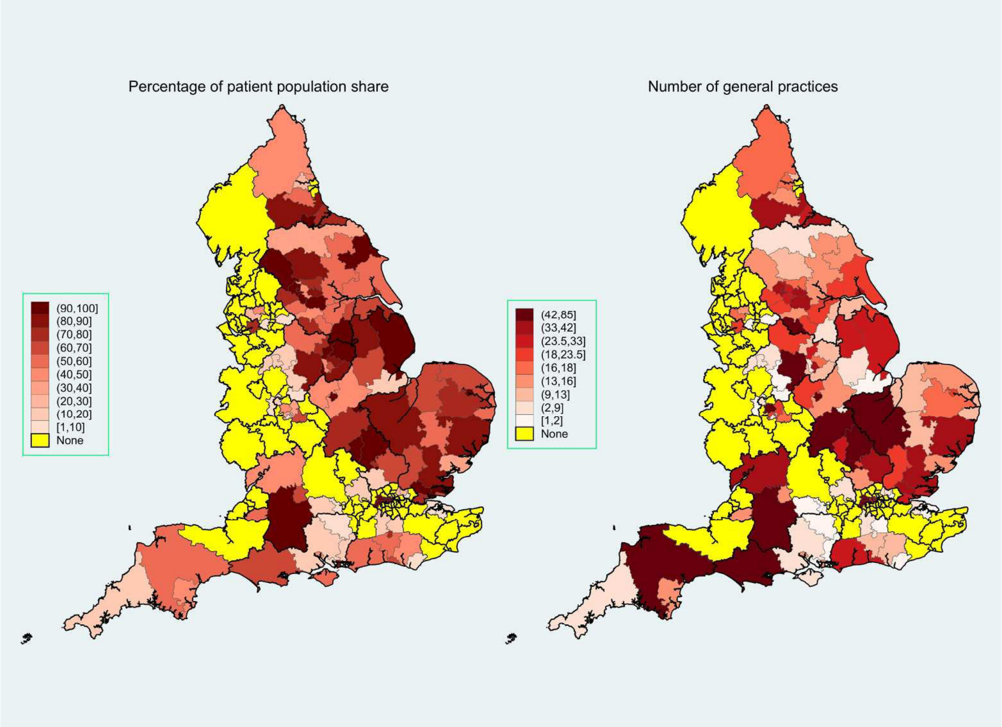

Figure 2: Spatial map at the Clinical Commissioning Group (CCG) level, September 2016: SystmOne

101x73mm (600 x 600 DPI)

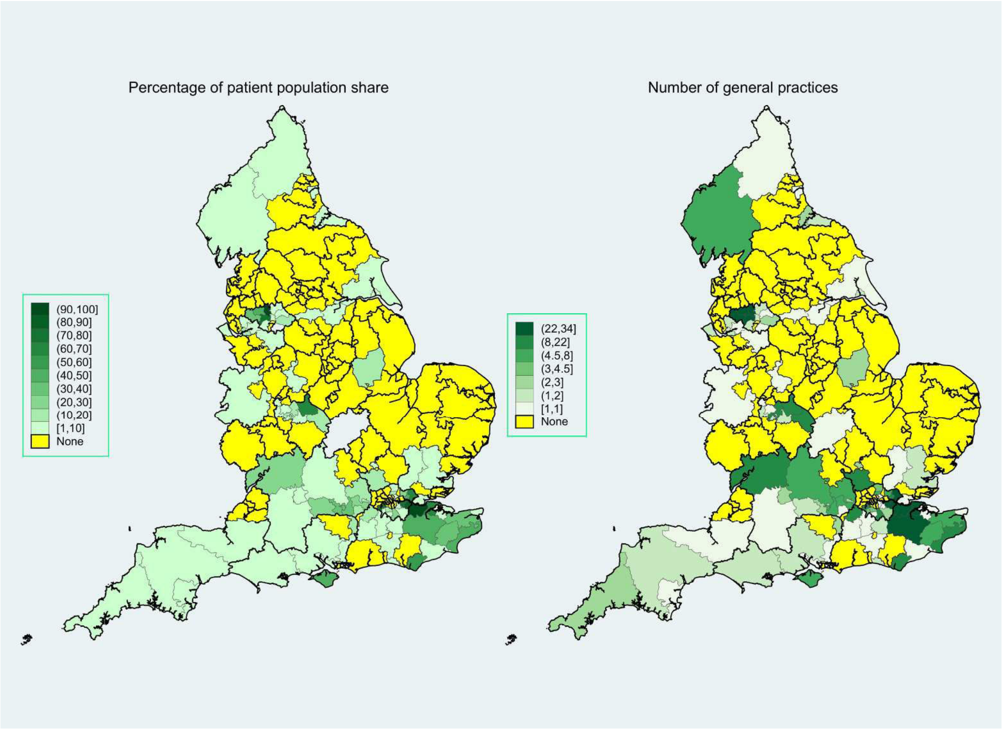

Figure 3: Spatial map at the Clinical Commissioning Group (CCG) level, September 2016: Vision

101x73mm (600 x 600 DPI)

ew only

STROBE 2007 (v4) Statement—Checklist of items that should be included in reports of *cross-sectional studies*

Section/Topic	Item #	Recommendation	Reported on page #
**Title and abstract**	1	(*a*) Indicate the study's design with a commonly used term in the title or the abstract	2
		(*b*) Provide in the abstract an informative and balanced summary of what was done and what was found	2
**Introduction**			
Background/rationale	2	Explain the scientific background and rationale for the investigation being reported	4-5
Objectives	3	State specific objectives, including any prespecified hypotheses	5
**Methods**			
Study design	4	Present key elements of study design early in the paper	5-6
Setting	5	Describe the setting, locations, and relevant dates, including periods of recruitment, exposure, follow-up, and data collection	5-6
Participants	6	(*a*) Give the eligibility criteria, and the sources and methods of selection of participants	5-6
Variables	7	Clearly define all outcomes, exposures, predictors, potential confounders, and effect modifiers. Give diagnostic criteria, if applicable	5-6
Data sources/ measurement	8*	For each variable of interest, give sources of data and details of methods of assessment (measurement). Describe comparability of assessment methods if there is more than one group	5-6
Bias	9	Describe any efforts to address potential sources of bias	NA
Study size	10	Explain how the study size was arrived at	5-6
Quantitative variables	11	Explain how quantitative variables were handled in the analyses. If applicable, describe which groupings were chosen and why	6
Statistical methods	12	(*a*) Describe all statistical methods, including those used to control for confounding	NA
		(*b*) Describe any methods used to examine subgroups and interactions	NA
		(*c*) Explain how missing data were addressed	NA
		(*d*) If applicable, describe analytical methods taking account of sampling strategy	NA
		(*e*) Describe any sensitivity analyses	NA
**Results**			

For peer review only

Participants	13*	(a) Report numbers of individuals at each stage of study—eg numbers potentially eligible, examined for eligibility, confirmed eligible, included in the study, completing follow-up, and analysed	6
		(b) Give reasons for non-participation at each stage	NA
		(c) Consider use of a flow diagram	NA
Descriptive data	14*	(a) Give characteristics of study participants (eg demographic, clinical, social) and information on exposures and potential confounders	6 and 12
		(b) Indicate number of participants with missing data for each variable of interest	6
Outcome data	15*	Report numbers of outcome events or summary measures	6 and 12
Main results	16	(*a*) Give unadjusted estimates and, if applicable, confounder-adjusted estimates and their precision (eg, 95% confidence interval). Make clear which confounders were adjusted for and why they were included	NA
		(*b*) Report category boundaries when continuous variables were categorized	12
		(*c*) If relevant, consider translating estimates of relative risk into absolute risk for a meaningful time period	NA
Other analyses	17	Report other analyses done—eg analyses of subgroups and interactions, and sensitivity analyses	NA
**Discussion**			
Key results	18	Summarise key results with reference to study objectives	6-7
Limitations	19	Discuss limitations of the study, taking into account sources of potential bias or imprecision. Discuss both direction and magnitude of any potential bias	7
Interpretation	20	Give a cautious overall interpretation of results considering objectives, limitations, multiplicity of analyses, results from similar studies, and other relevant evidence	7-8
Generalisability	21	Discuss the generalisability (external validity) of the study results	7-8
**Other information**			
Funding	22	Give the source of funding and the role of the funders for the present study and, if applicable, for the original study on which the present article is based	9

*Give information separately for cases and controls in case-control studies and, if applicable, for exposed and unexposed groups in cohort and cross-sectional studies.

Note: An Explanation and Elaboration article discusses each checklist item and gives methodological background and published examples of transparent reporting. The STROBE checklist is best used in conjunction with this article (freely available on the Web sites of PLoS Medicine at http://www.plosmedicine.org/, Annals of Internal Medicine at http://www.annals.org/, and Epidemiology at http://www.epidem.com/). Information on the STROBE Initiative is available at www.strobe-statement.org.

For peer review only
